# Formulation Design and Optimization of Fast Disintegrating Lorazepam Tablets by Effervescent Method

**DOI:** 10.4103/0250-474X.73911

**Published:** 2010

**Authors:** S. B. Shirsand, Sarasija Suresh, L. S. Jodhana, P. V. Swamy

**Affiliations:** Department of Pharmaceutics, H.K.E. Society’s College of Pharmacy, Sedam Road, Gulbarga - 585 104, India; 1 Department of Pharmaceutics Al-Ameen College of Pharmacy, Near Lal Bagh Main Gate, Hosur Road, Bangalore-560 027, India

**Keywords:** 3^2^ full factorial design, citric acid and tartaric acid, crospovidone, fast disintegrating tablets, lorazepam, sodium bicarbonate

## Abstract

Fast disintegrating tablets of lorazepam were prepared by effervescent method with a view to enhance patient compliance. A 3^2^ full factorial design was applied to investigate the combined effect of two formulation variables: amount of crospovidone and mixture of sodium bicarbonate, citric acid and tartaric acid (effervescent material) on *in vitro* dispersion time. Crospovidone (2-8% w/w) was used as superdisintegrant and mixture of sodium bicarbonate, citric acid and tartaric acid (6-18% w/w) was used as effervescent material, along with directly compressible mannitol to enhance mouth feel. The tablets were evaluated for hardness, friability, thickness, drug content uniformity and *in vitro* dispersion time. Based on *in vitro* dispersion time (approximately 13 s); the formulation containing 8% w/w crospovidone and 18% w/w mixture of sodium bicarbonate, citric acid and tartaric acid was found to be promising and tested for *in vitro* drug release pattern (in pH 6.8 phosphate buffer), short-term stability and drug-excipient interaction. Surface response plots are presented to graphically represent the effect of independent variables (concentrations of crospovidone and effervescent material) on the *in vitro* dispersion time. The validity of the generated mathematical model was tested by preparing two extra-design check point formulations. The optimized tablet formulation was compared with conventional marketed tablet for drug release profiles. This formulation showed nearly eleven-fold faster drug release (t_50%_ 2.8 min) compared to the conventional commercial tablet formulation (t_50%_ >30 min). Short-term stability studies on the formulation indicated that there were no significant changes in drug content and *in vitro* dispersion time (*P*<0.05).

The major problem faced by many patients with conventional tablet dosage form is difficulty in swallowing. This problem is more apparent when drinking water is not easily available to the patient taking medicine. Hence, patients may not comply with prescription, which results in high incidence of ineffective therapy[1]. The fast dissolving drug delivery system is rapidly gaining acceptance as an important novel drug delivery system. This delivery system emerged from the desire to provide patient with more convenient medication, with better patient compliance than with conventional tablet dosage form. Bioavailability of the drug from this delivery system is significantly greater than from conventional tablets[2–4]. Lorazepam is a benzodiazepine derivative with marked antiepileptic properties. It may be used in the treatment of all types of epilepsy and seizures[5]. Epileptic patients have to strictly adhere to the dosage schedule and any non-compliance of dosage administration may lead to sub-therapeutic levels of drug in the systemic circulation, and hence recurrence of seizures. Since a fast dissolving tablet (FDT) of the drug rapidly disintegrates in the mouth without need of water, such a dosage form will definitely enhance the patient compliance, especially, during travelling or in situations where water is not easily available. Hence, there is an obvious need for the development of fast dissolving tablets to overcome patient non-compliance. Further effervescent tablets have several advantages; (a) they are outstanding due to ease of administration and improved absorption of the active drug through previous dissolution in a buffered medium, (b) effervescent systems can buffer the aqueous solution of drug, so that the stomach pH increases leading to prevention of degradation or inactivation of the active ingredient. This buffering effect (via carbonation) induces the stomach to empty quickly, usually within 20 min into small intestine and results in maximum absorption of active ingredient, (c) effervescent tablets are advantageous since the drug product is already in solution at the time it is consumed, making the absorption faster and more complete when compared to conventional tablet, (d) they dissolve fully in a buffered solution. Reduced localized contact in upper GIT leads to less irritation and greater tolerability. Buffering also prevents gastric acids from interacting with drugs themselves, which can be a major cause of stomach and esophageal upsets, (e) they retain their palatability after lengthy storage; moreover, they produce fizzy tablets, which may have better consumer appeal than the traditional dosage forms, (f) excellent stability that is inherent to effervescent formulations, particularly surpassing liquid forms, (g) drugs delivered using effervescent technology have predictable and reproducible pharmacokinetic profiles that are more consistent than tablets or capsules, (h) effervescent components aid in improving the therapeutic profiles of active ingredients. They also help in solubilization of poorly soluble drugs, (i) effervescence induces penetration enhancement of broad range of compounds ranging in size, structure and other physiological properties. Effervescent blend can be used to obtain programmed drug delivery, (j) in remote areas, especially where parenteral forms are not available due to prohibitive cost, lack of qualified medical staff, effervescent tablets could become an alternative, e.g., the use of chloroquine phosphate effervescent tablets for treating malaria and viral fever and (k) to solve the problems of physicochemical stability and high cost of transporting syrups, effervescent tablets provide a realistic solution. Aim of the present study was to develop and optimize such a FDT for lorazepam using simple and cost-effective methodology.

## MATERIALS AND METHODS

Lorazepam and crospovidone (CP) were gift samples from Centaur Chemicals Pvt. Ltd., Chickboli, India, and Wockhardt Research Centre, Aurangabad, India, respectively. Directly compressible mannitol (Pearlitol SD200), sodium stearyl fumarate (SSF) were generous gifts from Strides Arco Labs, Bangalore, Glenmark Ltd., Nashik and Alkem Labs Pvt Ltd, Mumbai, India. All other chemicals used were of analytical reagent grade.

### Preparation of fast disintegrating tablets of lorazepam:

Fast disintegrating tablets of lorazepam were prepared by effervescent method[6] according to the formulae given in Table 1. Lorazepam, mannitol, pineapple flavour, aspartame and cros-povidone were accurately weighed and sifted through sieve No. 44. Sodium bicarbonate and anhydrous citric acid and tartaric acid were pre-heated at a temperature of 80° to remove absorbed/ residual moisture and were thoroughly mixed in a mortar to get a uniform powder and then added to other ingredients. The ingredients after sifting through sieve No. 44 were thoroughly mixed in geometrical order. The blend thus obtained was directly compressed at 8 mm size to get a tablet of 150 mg weight.

**TABLE 1:** FACTORIAL DESIGN FORMULATIONS OF LORAZEPAM PREPARED BY EFFERVESCENT METHOD

Ingredients (mg/Tablet)	Formulation code										
	EF_1_	EF_2_	EF_3_	EF_4_	EF_5_	EF_6_	EF_7_	EF_8_	EF_9_	C_1_	C_2_
Lorazepam	1.0	1.00	1.00	1.00	1.00	1.00	1.00	1.00	1.00	1.00	1.00
Crospovidone	3.0	3.00	3.00	7.50	7.50	7.50	12.00	12.00	12.00	5.25	9.75
Sodium bicarbonate	9.0	18.00	27.00	9.00	18.00	27.00	9.00	18.00	27.00	13.50	22.50
Citric acid+ tartaric acid	9.0	18.00	27.00	9.00	18.00	27.00	9.00	18.00	27.00	13.50	22.50
Aspartame	1.5	1.50	1.50	1.50	1.50	1.50	1.50	1.50	1.50	1.50	1.50
Flavour	1.5	1.50	1.50	1.50	1.50	1.50	1.50	1.50	1.50	1.50	1.50
Talc	3.0	3.00	3.00	3.00	3.00	3.00	3.00	3.00	3.00	3.00	3.00
Sodium stearyl fumarate	1.5	1.50	1.50	1.50	1.50	1.50	1.50	1.50	1.50	1.50	1.50
Mannitol SD200	120.5	102.5	84.5	116.0	98.0	80.0	111.5	93.5	75.5	109.2	86.75
Total	150.0	150.0	150.0	150.0	150.0	150.0	150.0	150.0	150.0	150.0	150.0

EF=Formulations prepared by effervescent method using cropsovidone as super-disintegrant at three different levels and mixture of sodium bicarbonate, citric acid and tartaric acid as effervescent material. C_1_ and C_2_ are extra design check-point formulations

### Evaluation of fast dissolving tablets:

To determine weight variation, twenty tablets were selected randomly from each formulation and were weighed individually using a Shimadzu digital balance (BL-220H). The individual weights were compared with the average weight for obtaining the weight variation[7]. Ten tablets from each formulation were selected randomly and their thickness was measured with a screw gauge for calculating thickness variation. Hardness of the tablets was measured using a Monsanto Hardness Tester (Pharmalab, Ahmedabad, India) and friability of a sample of twenty fast dissolving tablets was measured using a USP type-II Roche friabilator (Pharmalab, Ahmedabad, India). Pre-weighed tablets were placed in a plastic chambered friabilator attached to a motor revolving at a speed of 25 rpm for 4 min. The tablets were then dusted, reweighed and percentage weight loss (friability) was calculated (Table 2 and 3).

**TABLE 2:** FORMULATION AND EVALUATION OF 3^2^ FULL FACTORIAL DESIGN

Formulation code	Variable levels in coded form^*^		In vitro dispersion time
	X_1_	X_2_	
EF_1_	-1	-1	57.19
EF_2_	-1	0	53.04
EF_3_	-1	+1	43.64
EF_4_	0	-1	44.51
EF_5_	0	0	32.71
EF_6_	0	+1	22.35
EF_7_	+1	-1	25.09
EF_8_	+1	0	13.56
EF_9_	+1	+1	13.18
C_1_	-0.5	-0.5	42.24
C_2_	+0.5	+0.5	31.13

Where-1=3.0 mg, 0=7.5 and +1=12 mg (X_1_ is the amount of crospovidone). Where -1=9.0 mg of NaHCO_3_ and 9.0 mg citric acid and tartaric acid, 0=18.0 mg of NaHCO_3_ and 18.0 mg citric acid and tartaric acid +1=12 mg of NaHCO_3_ and 12 mg citric acid and tartaric acid (X_2_ is the amount of effervescent materials). -0.5= 5.25 mg (X_1_ is the amount of crospovidone and 13.5 mg of NaHCO_3_ and 13.5 mg of citric acid + tartaric acid (X_2_ is the amount of effervescent material) +0.5=9.75 mg (X_1_ is the amount of crospovidone) and 22.5 mg of NaHCO_3_ and 22.5 mg citric acid and tartaric acid (X_2_ is the amount of effervescent material)

**TABLE 3:** EVALUATION OF FACTORIAL DESIGN FDT FORMULATIONS

Parameters	Formulation code											
	EF_0_	EF_1_	EF_2_	EF_3_	EF_4_	EF_5_	EF_6_	EF_7_	EF_8_	EF_9_	C_1_	C_2_
Hardness* (kg/cm^2^)± SD	3.60±0.28	3.16±0.57	3.33±0.66	3.30±0.26	3.43±0.05	3.50±0.10	3.36±0.32	3.50±0.10	3.36±0.32	3.33±0.32	3.00±0.03	3.36±0.032
Thickness (mm)	2.4	2.74	2.85	2.80	2.78	2.90	2.90	2.56	2.54	2.70	2.90	2.85
Friability (%)	0.52	0.50	0.41	0.42	0.51	0.42	0.43	0.51	0.43	0.40	0.43	0.42
*In vitro* dispersion time* (sec) ±SD	180.27±0.14	57.19±0.87	53.04±1.23	43.64±1.29	44.51±3.40	32.71±1.32	22.35±1.46	25.09±1.62	13.56±0.65	13.18±1.03	42.24±0.74	31.13±2.72
Drug content* (%)±SD	101.74±1.25	104.23±1.87	103.59±0.60	104.28±0.66	102.74±2.15	103.36±1.21	102.74±2.15	103.25±1.12	103.36±1.21	104.28±0.66	103.54±0.61	105.37±0.67
Weight variation	146-152 mg (within the IP limits of ±7.5%)											

* Average of three determinations. Formulation EF_9_ was selected as the best and used in further studies

Drug content uniformity was determined by weighing ten tablets, pulverizing to a fine powder. A quantity of powder equivalent to 1 mg of lorazepam was extracted into methanol and the solution was filtered through a 0.22 µm membrane filter disc, (Millipore Corporation). The lorazepam content was determined by measuring the absorbance at 231 nm (UV/Vis spectrophotometer, Shimadzu 1700) after appropriate dilution with methanol. The drug content was determined using standard calibration curve. The mean percent drug content was calculated as an average of three determinations[8].

*In vitro* dispersion time was determined by placing one tablet in a beaker containing 10 ml of pH 6.8 phosphate buffer at 37±0.5° and the time required for complete dispersion was determined[9]. Wetting time was determined by carefully placing a tablet on to a twice folded circular tissue paper placed in a Petri-dish having internal diameter of 5cm containing 6 ml of water. The time required for water to reach the upper surface of the tablet and to completely wet it was noted as the wetting time. The weight of the tablet prior to placing in the Petri-dish was noted (w_b_) using a Shimadzu balance. The wetted tablet was removed and reweighed (w_a_). Water absorption ratio (R) was then determined according to the following equation; R=100×(w_a_ – w_b_)/w_b,_ where, w_b_ and w_a_ were tablet weights before and after water absorption, respectively.

### *In vitro* drug release study:

*In vitro* dissolution studies of the optimized fast dissolving tablets of lorazepam and commercial conventional tablet was performed according to USP XXIII Type-II dissolution apparatus (Electrolab, model TDT-06N) employing a paddle stirrer at 50 rpm using 900 ml of pH 6.8 phosphate buffer at 37±0.5° as dissolution medium[10]. One tablet was used in each test. Aliquotes of the dissolution medium (5 ml) were withdrawn at specific time intervals (2, 4, 6, 8, 10, 15 and 30 min) and replaced with the equal volume of fresh medium. The samples were filtered through 0.22 µm membrane filter disc and analyzed for drug content by measuring the absorbance at 233 nm. Drug concentration was calculated from the standard calibration curve and expressed as cumulative percent drug dissolved. The release studies were performed in replicates of six.

### Stability testing:

Accelerated stability studies on formulation EF_9_ were carried out by storing 15 tablets in an amber coloured rubber stoppered vials at 40°/75% RH over a period of 3 mo. At intervals of one month, the tablets were visually examined for any physical changes and changes in drug content. At the end of 3 mo period, the formulation was also subjected to dissolution studies.

### Optimization of the formulation using 3^2^ full factorial design:

Based on the results of the preliminary trial formulations of 13 batches of three different combinations of effervescent materials, a 3^2^ full factorial design was used for simultaneous evaluation of two formulation variables and their interaction, each at three levels and trials were performed at all nine possible combinations[11]. The best combination (blend of sodium bicarbonate-citric acid and tartaric acid)[12] of effervescent material was used in the optimization of the formulations. The effect of the independent variables, viz., crospovidone (X_1_) and blend of sodium bicarbonate-citric acid and tartaric acid (X_2_) on the dependent variable *in-vitro* dispersion time (Y_1_) was evaluated.

## RESULTS AND DISCUSSION

Lorazepam FDT were prepared by effervescent method; formulation optimization was done using a 3^2^ full factorial design employing crospovidone as super-disintegrant and blend of sodium bicarbonate-citric acid and tartaric acid as an effervescent material, along with directly compressible mannitol (Pearlitol SD 200), which was used to enhance the mouth feel, based on the results of preliminary trial formulations. A total of nine formulations, a control formulation (EF_0_ without super disintegrant) and two extra-design check point formulations (C_1_ and C_2_ to check validity of the developed polynomial equation), were designed.

Powder blends were evaluated for the flow parameters such as angle of repose, tapped density, bulk density and Carr’s index. As the material was free flowing (angle of repose values were found to be <30°and Carr’s index <15%).

The tablets were evaluated for weight variation, uniformity of drug content, hardness, friability, *in vitro* dispersion time, *in vitro* dissolution studies, tablets obtained were of uniform weight (due to uniform die fill), with acceptable variation as per IP specifications (±7.5%). Drug content was found to be in the range of 102-105%, which is within acceptable limits. Hardness of the tablets was found to be 3.0 to 3.5 kg/cm^2^. Friability below 1% was an indication of good mechanical resistance of the tablets. Formulation EF_9_ was found to be promising and displayed an *in vitro* dispersion time of 13 s, which facilitates faster dispersion in the mouth.

Based on the results of preliminary trail formulations of 13 batches, optimization of the FDT formulation has been done using a 3^2^ full factorial design (formulations EF_1_ to EF_9_). PCP Disso 2000 V3 software was used to develop a polynomial equation for the dependent variable *in vitro* dispersion time. Formulation EF_9_ containing 8% w/w crospovidone, 18% w/w mixture of sodium bicarbonate-citric acid and tartaric acid was found to be promising with an *in vitro* dispersion time of 13 s against 180 s displayed by control formulation (EF_0_), which does not contain the super-disintegrant crospovidone.

**Fig. 1:**
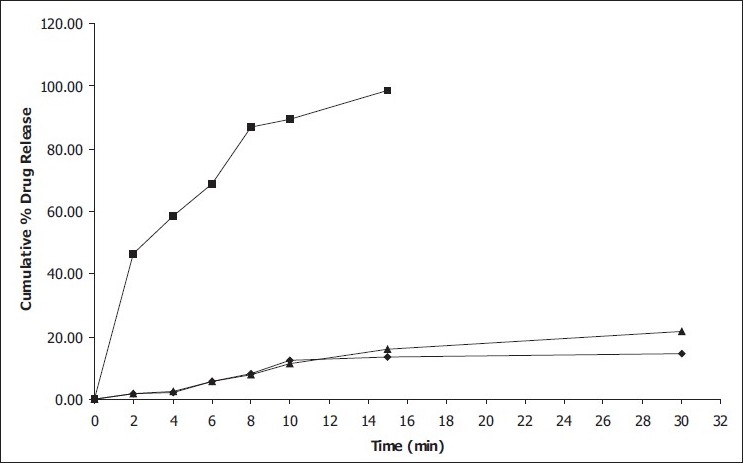
*In vitro* cumulative percent drug release versus time profile of promising lorazepam formulations Plot showing cumulative percent drug release in pH 6.8 phosphate buffer from control formulations EF_0_ (– ♦ –); promising EF_9_ formulation (– ■ –); conventional commercial tablet formulation CCF (– ♦ –)

*In vitro* dissolution parameters, including dissolution efficiency[13] of the promising formulation EF_9_, the control (EF_0_) and the commercial conventional tablet formulation (CCF) are shown in Table 4and the dissolution profiles, and dissolution parameters depicted in figs. 1 and 4 respectively. This data reveals that overall, the formulation EF_9_ has shown nearly eleven-fold faster drug release (t_50%_ 2.8 min) when compared to CCF (t_50%_ > 30 min).

**TABLE 4:** *IN VITRO* DISSOLUTION PARAMETERS IN PH 6.8 PHOSPHATE BUFFER

Formulation code	D_5_ (%)	D_10_ (%)	DE_5min_ (%)	DE_10min_ (%)	t_50%_ (min)	t_70%_ (min)	t_90%_ (min)
EF_0_	5.00	12.59	6.00	14.43	>30	>30	>30
EF_9_	62.00	89.00	13.93	33.02	2.80	6.10	9.0
CCF	4.00	11.38	10.00	19.45	>30	>30	>30

EF_0_ is control formulation, EF_9_ is promising fast disintegrating tablet formulation, CCF is conventional commercial tablet formulation, D_5_ is percent drug released in 5 min, D_10_ is percent drug release in 10 min, D_15_ is percent drug release in 15 min, DE_10min_ is dissolution efficiency at 10 min, t_50%_ is time for 50% drug dissolution, t_70%_ is time for 70% drug dissolution, t_90%_ is time for 90% drug dissolution

IR spectroscopic studies indicated that the drug is compatible with all the excipients. The IR spectrum of EF_9_ showed all the characteristic peaks of lorazepam, thus confirming that no interaction of drug occurred with the components of the formulation. Short-term stability studies of the above formulation indicated that there are no significant changes in drug content and *in vitro* dispersion time at the end of 3 mo period (*P*<0.05).

Polynomial equation for 3^2^ full factorial design with two independent variables i.e., proportion of crospovidone (X_1_) and proportion of sodium bicarbonate, citric acid and tartaric acid as effervescent material (X_2_), at three levels is[14] Y = b_0_ + b_1_ X_1_ + b_2_ X_2_ + b_12_ X_1_ X_2_ + b_11_ X_1_^2^ + b_22_ X_2_^2^, where Y is dependent variable, b_0_ arithmetic mean response of nine batches, and b_1_ estimated coefficient for factor X_1_. The main effects (X_1_ and X_2_) represent the average results of changing one factor at a time from its low to high value. The interaction term (X_1_ X_2_) shows how the response changes when two factors are simultaneously changed. The polynomial terms X_1_^2^ and X_2_^2^ are included to investigate non-linearity.

**Fig. 2:**
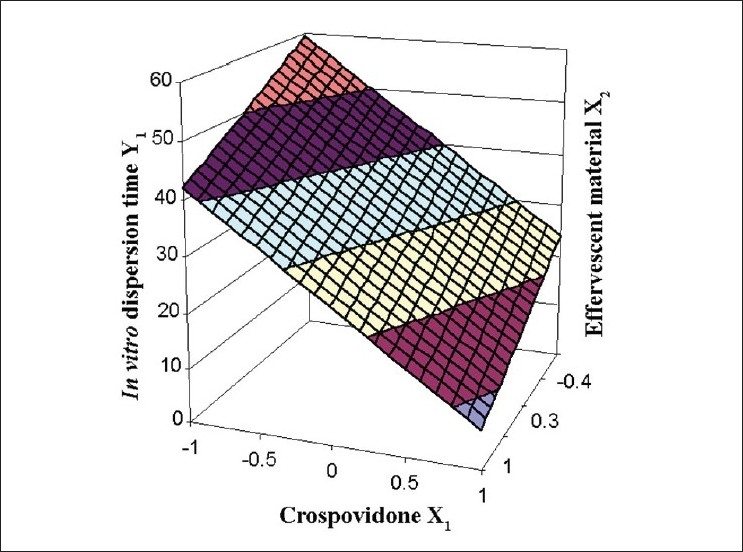
Response surface plot of factorial variables on *in vitro* dispersion time The shaded regions indicate the range of response variables, Y_1_ (*in vitro* dispersion time)

**Fig. 3:**
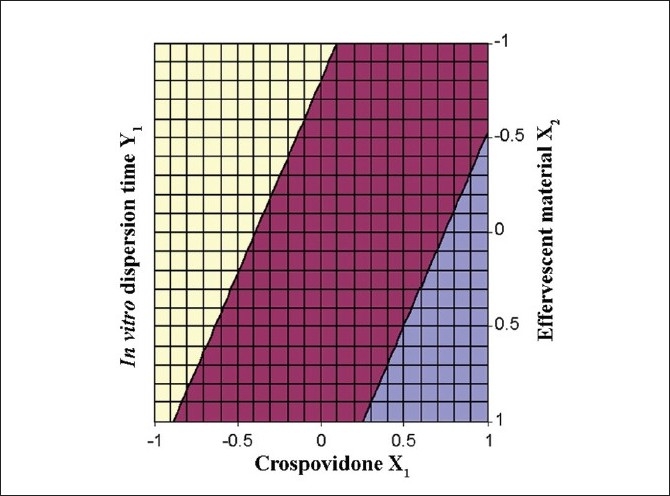
Contour plot of factorial variables on *in vitro* dispersion time The shaded regions indicate the range of response variables, Y_1_ (*in vitro* dispersion time)

The Eqn. derived for *in vitro* dispersion time of the factorial formulations is Y_1_ = 33.11–8.83 X_1_ –4.33 X_2_. The negative sign for coefficients of X_1_ and X_2_ indicate that as the concentration of disintegrants increases, *in vitro* dispersion time decreases. The data clearly indicates that the *in vitro* dispersion time values are strongly dependent on the selected independent variables. Validity of the above equation was verified by designing two extra design check point formulations (C_1_ and C_2_) and determining the *in vitro* dispersion time. The *in vitro* dispersion time values predicted from the equation for these formulations are 44.73 and 17.73 s, where those observed from experimental results are 46.27 and 19.95 s, respectively. The closeness of the predicted and observed values for C_1_ and C_2_ in the method indicates validity of derived equation for the dependent variable (*in vitro* dispersion time). The computer generated response surface and contour plots for the dependent variable are shown in fig. 2 and 3, respectively.

**Fig. 4:**
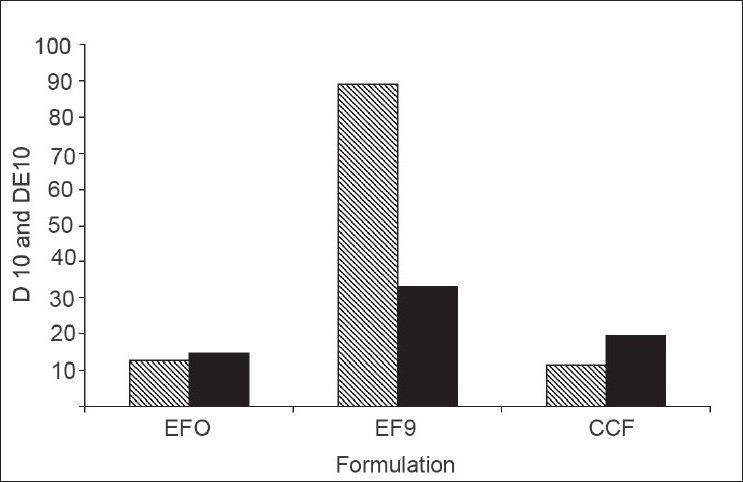
Comparative *in vitro* drug release and dissolution efficiency D_10_ (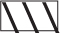) cumulative percent drug release in 10 min; DE_10_ min (■ Dissolution efficiency after 10 min; EFO= control formulation; EF_9_= promising formulation and CCF = conventional commercial tablet

The results a 3^2^ full factorial design reveal that the amounts of crospovidone (X_1_) and effervescent material (X_2_) significantly affect the dependent variable (Y_1_), the *in vitro* dispersion time. It is thus concluded that, by adopting a systematic formulation approach, an optimum point can be reached in the shortest time with minimum efforts. Effervescent technique would be an effective approach compared with the use of more expensive adjuvants in the formulation of fast dissolving tablets with improved drug dissolution, patient compliance, convenience and acceptability.
